# Monitoring for PERV Following Xenotransplantation

**DOI:** 10.3389/ti.2024.13491

**Published:** 2024-10-07

**Authors:** Joachim Denner

**Affiliations:** Institute of Virology, Free University Berlin, Berlin, Germany

**Keywords:** virus safety of xenotransplantation, retroviruses, porcine endogenous retroviruses (PERVs), PERV-C, vaccine

## Abstract

Porcine endogenous retroviruses (PERVs) are integrated in the genome of all pigs. PERV-A, PERV-B and PERV-C can be released as infectious virus particles and PERV-A and PERV-B can infect human cells in culture. PERV-C does not infect human cells, but high-titer recombinant PERV-A/C can infect them. Retroviruses are able to induce immunosuppression and/or tumors in the infected host. Numerous methods have been developed to study PERV in donor pigs. No PERV infections were observed in infection experiments as well as in preclinical and clinical xenotransplantation trials. Despite this, several strategies have been developed to prevent PERV infection of the recipient. PCR-based and immunological methods are required to screen xenotransplant recipients. Since the proviruses are integrated into the pig genome, PERV infection has to be distinguished from microchimerism, e.g., the presence of pig cells in the recipient, which is common in xenotransplantation. Sensitive PCR methods using pig short interspersed nuclear elements (SINE) sequences allow to detect pig cells easily. Virus infection can also be detected by an increase of viral genomic or mRNA in human cells. The method of choice, however, is to screen for specific antibodies against PERV using different recombinant PERV proteins, purified viruses or peptides.

## Introduction

Xenotransplantation may be associated with the transmission of pathogenic pig viruses. The recent report on the first patient receiving a pig heart in Baltimore, Maryland, United States, underlined the importance of virus safety in the context of xenotransplantation. The porcine cytomegalovirus, which is correctly defined as a porcine roseolovirus (PCMV/PRV), was not detected in the xenotransplant due to inappropriate detection methods, but in the transplanted organ the virus was massively replicating due to the absence of the porcine immune system and this contributed to the cascade leading to the death of the patient [1]. Whereas PCMV/PRV and other viruses such as the zoonotic virus hepatitis E virus (HEV), which may induce a disease in the recipient, can be easily eliminated by selection of virus-negative animals, early weaning, colostrum-deprivation, Cesarean delivery or embryo transfer [2], this is not possible with PERVs, since their proviruses are integrated in the genome of all pigs. Therefore, PERVs pose a special risk for xenotransplantation using pig cells or organs: Many retroviruses are able to induce immunodeficiencies and tumors in the infected host. Not only the human immunodeficiency virus (HIV) induces severe immunodeficiencies, but also viruses closely related to PERV such as the murine leukemia virus (MuLV), the feline leukemia virus (FeLV) and the koala retrovirus (KoRV) induce immunodeficiencies in addition to leukemias and lymphomas in the infected host. Since there are excellent reviews available, describing the biology, the detection of PERV in donor pigs and the strategies how to prevent PERV transmission to the recipients [3–6], I will give only a short summary on these topics and concentrate on monitoring for PERV infection following xenotransplantation.

## Biology of Pervs

Retroviruses are characterized by their ability to integrate their RNA genome using the viral enzymes reverse transcriptase and integrase as a DNA copy into the genome of the infected cell. This integrated DNA copy is called provirus. Endogenous retroviruses are the result of integration of proviruses into the oocyte or the sperm cell and consequently, they are present in all cells of the developing organism. PERV-A and PERV-B are present in the genome of all pigs, whereas PERV-C is present in most, but not all pigs. PERV-A and PERV-B are able to infect human cells in culture and therefore pose a risk for xenotransplantation, whereas PERV-C infects only pig cells [5]. However, PERV-C can recombine with PERV-A and acquire so the receptor binding site for the receptor of PERV-A. PERV-A/C can therefore infect human cells and replicate with higher titers compared to the paternal PERV-A. PERV-A and PERV-B infect mainly human tumor cells [7–9], reports concerning the infection of primary cells are rare [9, 10].

## Detection in Donor Pigs

PCR and real-time PCR methods can be used to detect PERV proviruses in the genome of pigs. Droplet digital PCR (ddPCR) allows to quantify the average number of proviruses in the genome of one cell. The copy number depends on the pig breed. In European pigs the copy number is around 60, in Asian pigs the copy number is slightly lower (for review see [11, 12]). To analyze the expression of PERVs at the mRNA level, reverse transcriptase (RT) real-time PCR may be used. To detect the expression at the protein level, immunofluorescence, immunoperoxidase assay or immunohistochemistry using specific sera against viral proteins may be applied. Electron microscopy and measurement of RT activity can be applied to measure the release of virus particles. Infections assays can be used to detect infectious virus particles. PERVs able to infect pig cells will be detected if pig cells are used in these assays; PERVs able to infect human cells will be detected if human cells are used. Details of the mentioned detection methods are described in [13–21].

## Strategies to Prevent Transmission

Trans-species transmissions of retroviruses in the sense of infection are well known [2, 22], and the AIDS pandemic is the most disastrous example of a transmission of a zoonotic retrovirus to humans [23]. PERV itself is the result of trans-species transmission of retroviral sequences from rock hyrax, lesser Egyptian jerboa and rodents into pigs [24, 25]. Until now, PERV infections were not observed in all preclinical trials transplanting pig organs into NHPs [26–33]. No PERV infection was observed in first clinical trials transplanting pig cells and organs into human patients, too [26, 34–40]. Furthermore, PERV infection was not observed in infection experiments with small animals as well as with non-human primates under strong immunosuppression [33]. Despite this, numerous strategies to prevent transmission including selection of PERV-C-free animals using PERV-C-specific detection methods to prevent recombination with PERV-A, antiretroviral drugs which are partially also used against (HIV [41, 42], and RNA interference using siRNA [43–47] were developed. Furthermore, vaccines on the basis of neutralizing antibodies against the transmembrane envelope protein p15E and the surface envelope protein gp70 of PERV were generated [48–50]. However, these vaccines could not be tested due the absence of an animal model of PERV infection. Therefore, similar vaccines were developed against the closely related FeLV and it was shown that the vaccine prevented leukemia outbreak in cats infected with FeLV after immunization [51]. Best results were obtained by genome editing. After the failure to inactivate all PERVs using zinc finger nuclease [52], the application of CRISPR/Cas resulted in successful inactivation of all integrated PERVs by deletions in the reverse transcriptase (RT) gene *in vitro* [53] and *in vivo* [54]. However, until now it is unclear whether this inactivation is necessary because - as described in the beginning of this chapter – there is no evidence of PERV transmission to date. Furthermore, there may be off target effects of CRISPR/Cas, and it will be difficult to breed these animals to large colonies. It was shown that CRISPR/Cas treated pig cells are still able to release intact virus particles [55], which, however, should contain viral genomic RNA with an inactivated RT. It is likely that these particles can perform entry into human cells because they carry functional envelope proteins in their envelope. However, due to the inactivated RT they cannot integrate into the genome of the target cell. Since some human cells express RT, either from LINE sequences [56] or from human endogenous retroviruses (HERVs) [57], it cannot be excluded that these RT rescue PERV facilitating reverse transcription and integration. However, it is unlikely that the inactivating mutation in the PERV RT can be repaired or that a recombination between LINE or HERV-RT and PERV-RT is taking place to rescue the virus completely.

## Detection in the Recipient

**FIGURE 1:**
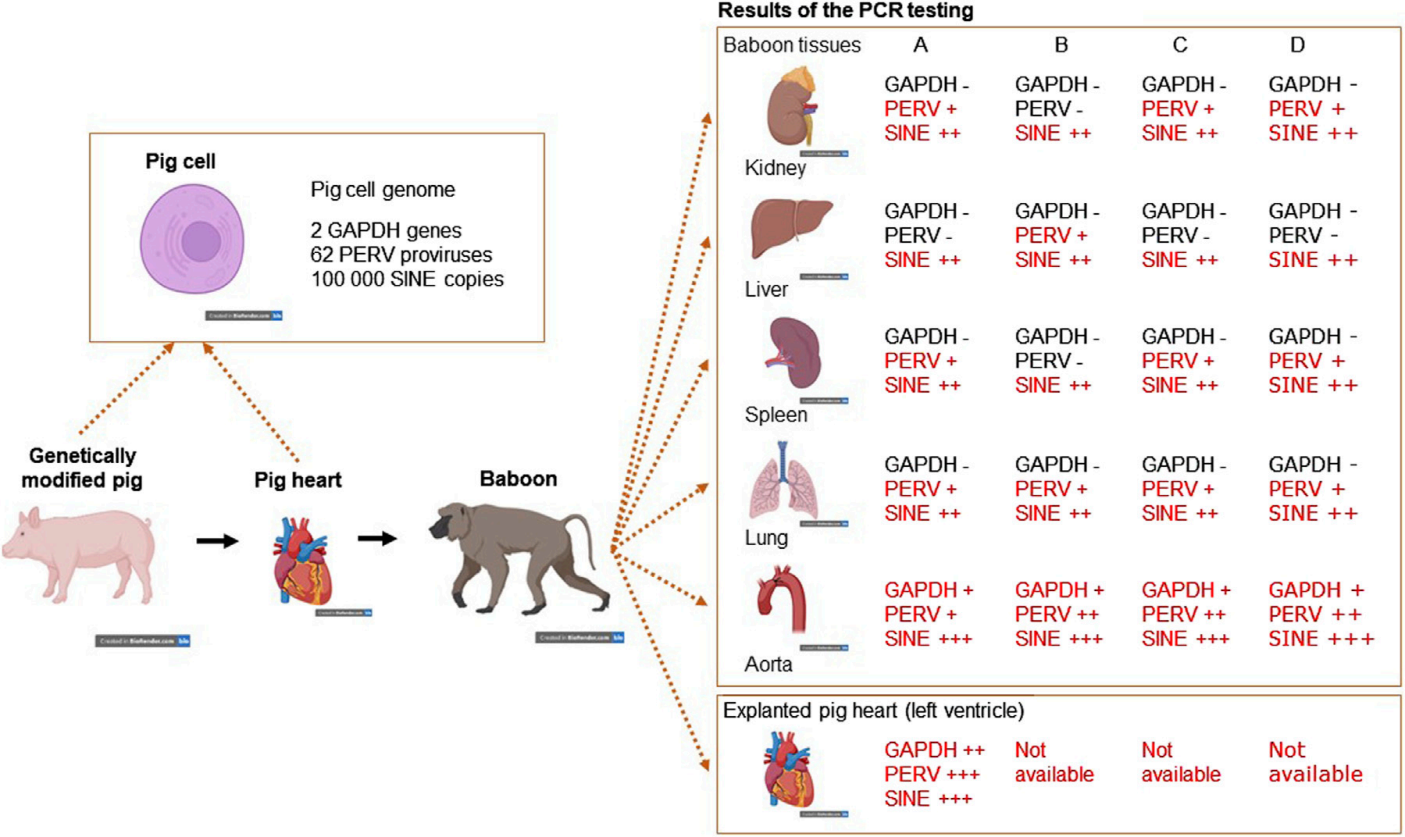
Detection of microchimerism in organs from four baboons (baboon A – survival time 195 days, baboon B – 194 days, baboon C – 26 days, and baboon D – 50 days) after transplantation of a heart from a genetically modified pig. DNA from kidney, liver, spleen, lung and aorta as well as from the explanted pig heart were screened for the presence of porcine GAPDH (2 copies in the pig genome), PERV (approximately 60 copies) and SINE (more than 100,000 copies) using real-time PCRs. Whereas GAPDH as pig marker was found only in the aorta, which is partially of pig origin and in the explanted heart, PERV was found in all organs with exception of the livers from baboons A. C and D. The SINE PCR detected pig cells in all organs analyzed, indicating the presence of microchimerism (This is the Graphical Abstract of publication [59]).

To screen PERV in the recipient of pig xenotransplants, PCR-based and immunological methods can be used. The use of PCR methods to screen for PERV proviral sequences is difficult, because these sequences are part of the pig genome. Pig cells will be found in all organs of the recipient and will interfere with this testing. The presence of donor cells in the recipient, called microchimerism, is common in xenotransplantation as well as in allotransplantation and pregnancy (for review see [58]). To make sure that the detected proviruses are part of the genome of pig cells, a PCR was developed which detects pig sequences, the so called short interspersed nuclear elements (SINE) sequences [59]. These sequences are found more than 100,000 times in the genome of pigs and this high copy number makes it easy to detect pig cells (Figure 1). When we screened baboons for PERV, PERV sequences were found in most of the organs tested (there are around 60 PERV copies in the pig genome), GAPDH sequences were rarely found (there are only 2 copies in the pig genome), but SINE sequences (more than 100,000 copies per genome) were found in all baboon organs tested, indicating microchimerism (Figure 1) [59]. Another possibility is to screen for increasing amounts of viral genomic or viral mRNA using a RT real-time PCR or sequencing of RNA, indicating virus replication. Another very effective way of testing for PERV would be looking for spliced env mRNA, which is a prerequisite for the translation of the Env protein and particle release [60, 61]. However, there is the possibility that viral RNA will be produced in pig cells expressing PERV or even releasing PERV. To demonstrate an infection, viral RNA has to be localized in human cells.

**FIGURE 2:**
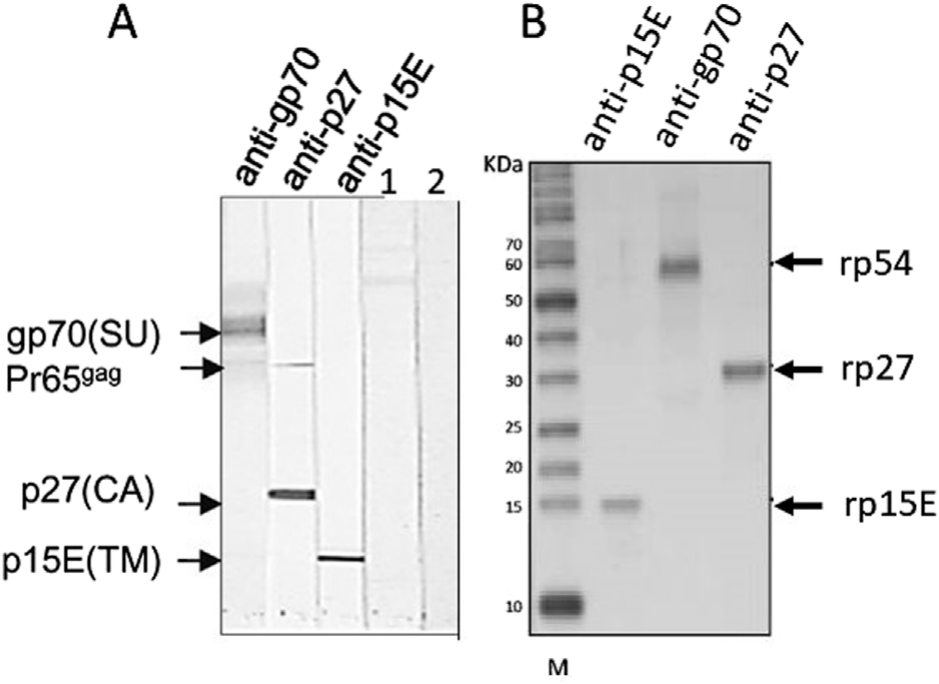
**(A)** Western blot analysis using gradient purified virus particles separated on an SDS-PAGE and goat sera raised against the recombinant surface envelope protein gp70 (SU) of PERV, recombinant capsid p27 (CA) and recombinant transmembrane envelope protein p15E (TM). The sera detect the corresponding viral proteins as well as the precursor molecule of Gag, Pr65^gag^. In the lanes 1 and 2 sera from patients receiving pig islet cells in the Argentina trial were tested negative (published in [34], with kind permission of Elsevier, 240606-011543). **(B)** Western blot analysis using recombinant rp54 as unglycosylated gp70, rp27Gag and rp15E, which is p15E without the membrane spanning region and the endodomain, and the goat sera as in A. M, marker. (published in [63], with kind permission of Elsevier).

Having in mind these difficulties, the best method to detect PERV infection in the recipient is to detect antibodies against PERV as an indirect sign of virus infection. Antibody screening is a common method to detect retrovirus infection and is widely used to screen for an infection with HIV [62]. There are two main methods to detect PERV-specific antibodies, ELISA or Western blot analysis. Recombinant PERV proteins or purified virus particles can be used as antigens in Western blot analyses [19, 28, 34, 38] (Figures 2A, B). The advantage of Western blot analyses when using recombinant proteins is that the specific band can be accurately identified based on size, whereas false-positive reactions can be expected in ELISA when using recombinant proteins due to insufficient purity of the proteins produced in bacteria. Using lysates of highly purified virus preparations also allows to detect antibody responses against different viral proteins (Figure 2A). An ELISA can be performed using synthetic peptides corresponding to immunodominant epitopes of the viral proteins [19]. Such a immunodominant epitope was detected in the transmembrane envelope protein of several retroviruses [19]. It is very important to use two or three PERV proteins, for example the core protein p27Gag, the surface envelope protein gp70 and the transmembrane envelope protein p15E or corresponding synthetic peptides (Figure 2). It is not recommended to use p27Gag of PERV alone since there are a few human individuals who have antibodies against p27Gag of PERV, despite the fact that they are not infected [19]. The absence of antibodies against other PERV proteins indicates that they are not infected. It remains unclear whether the response against p27Gag in these few individuals is due to cross-reactive antibodies directed against an auto-antigen, a parasite-derived antigen or a related retrovirus [64–67]. False positive antibodies against the core protein p24 of HIV-1 were also common, for example in patients with systemic lupus erythematosus [68, 69].

There is one additional question: Are immunosuppressed individuals who received a pig organ able to mount an antibody response? Since immunosuppressed patients after allotransplantation are able to mount sufficient immune responses after vaccination, it is likely that xenotransplant patients can produce antibodies against PERV proteins [70, 71].

## Conclusion

**FIGURE 3:**
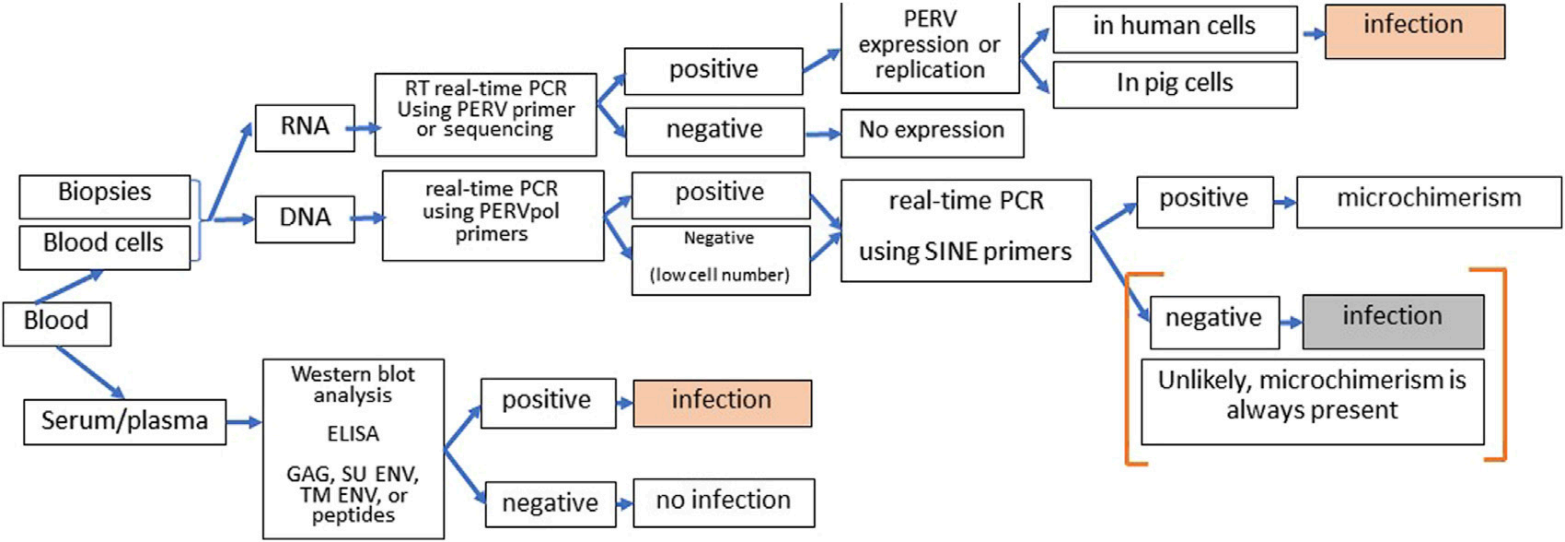
Strategy of screening for PERV infection in xenotransplantation recipients. DNA and RNA from blood cells or biopsies can be tested by PCR or RT-PCR for the presence of PERV proviruses and PERV expression. Since microchimerism is always present in xenotransplanted individuals, detection of PERV sequences in the DNA will always be associated with pig cell. When increasing amounts of PERV genomic and mRNA were observed, it has to be assured that virus replication takes part in human cells to diagnose PERV infection of the recipient. The presence of antibodies against two or more PERV antigens (GAG, capsid protein p27; SU ENV, surface envelope protein gp70, TM ENV, transmembrane envelope protein p15E, or corresponding peptides) using Western blot assays or ELISA clearly indicates an PERV infection. Absence of immune reaction means absence of infection approximately 3 to 4 weeks before.

There are numerous assays available to screen for PERV infection in the recipient [72]. However, it is not easy since microchimerism, e.g., presence of pig cells in all organs of the recipient, complicates the proof. Therefore, it is important to distinguish between infection of human cells on one hand and proviruses in disseminated pig cells, which will always be present in xenotransplanted individuals, on the other hand. Highly sensitive SINE PCR will easily detect microchimerism. One further method is the detection of increasing amounts of viral genomic or mRNA indicating replication of PERV. However, to prove infection, it should be shown that this replication takes place in human and not in pig cells. The method of choice is the detection of antibodies against PERV using at least two or three recombinant viral proteins or corresponding synthetic peptides or purified virus particles in a Western blot assay or ELISA (Figure 3).

## Abbreviations

AIDS, acquired immune deficiency syndrome; CRISR/Cas, clustered regularly interspaced short palindromic repeats; CRISPR associated; ddPCR, droplet digital PCR; ELISA, enzyme-linked immunosorbent assay; FeLV, feline leukemia virus; GAPDH, glyceraldehyde-3-phosphate dehydrogenase; HERVs, human endogenous retroviruses; HEV, hepatitis E virus; HIV, human immunodeficiency virus; KoRV, koala retrovirus; LINE, long interspersed nuclear element; MuLV, murine leukemia virus; PCR, polymerase chain reaction; PERV, porcine endogenous retrovirus; PCMV/PRV, porcine cytomegalovirus/porcine roseolovirus; RT, reverse transcriptase; SINE, short interspersed nuclear elements.
